# Enhancing Energy Density of BaTiO_3_-Bi(M)O_3_@SiO_2_/PVDF Nanocomposites via Filler Component Modulation and Film Structure Design

**DOI:** 10.3390/nano15080569

**Published:** 2025-04-08

**Authors:** Jin Hu, Fangfang Liu

**Affiliations:** 1 State Key Laboratory of Fine Chemicals, Dalian University of Technology, Dalian 116024, China; 2 College of Science, National University of Defense Technology, Changsha 410073, China

**Keywords:** polymer nanocomposites, dielectric properties, breakdown strength, energy storage density

## Abstract

The low energy density (*U*_d_) of polymeric dielectrics is unfavorable for the integration and miniaturization of electronics, thus limiting their application prospects. Introducing high-*ε*_r_ (dielectric constant) ceramic nanofillers to polymer matrices is the most common strategy to enhance their *ε*_r,_ and hence their *U*_d_. By comparison, enhancing breakdown strength (*E*_b_) is a more effective strategy to enhance *U*_d_. Herein, 0.6BaTiO_3_-0.4Bi(Mg_0.5_Ti_0.5_)O_3_ and 0.85BaTiO_3_-0.15Bi(Mg_0.5_Zr_0.5_)O_3_ nanofibers coated with SiO_2_ were utilized as fillers in PVDF-based nanocomposites. The combination of experimental and simulation results suggests that the intrinsic properties of nanofillers are the determining factor of the *E*_b_ of polymer-based nanocomposites, and SiO_2_ coating and film structure design are effective strategies to enhance their *E*_b_, and consequently their *U*_d_. As a result, the sandwich-structured PVDF/6 wt% 0.85BaTiO_3_-0.15Bi(Mg_0.5_Zr_0.5_)O_3_@SiO_2_ nanofiber within PVDF/PVDF nanocomposite films achieved a maximum *U*_d_ of 11.1 J/cm^3^ at an *E*_b_ of 458 MV/m, which are 2.15 and 1.40 times those of pristine PVDF, respectively.

## 1. Introduction

Dielectric capacitors are one of the most important electric energy storage devices, and are present in almost every sort of electronic circuit [1]. With the ever-increasing demand for flexible, compact, and lightweight electronics, dielectric capacitors with high discharge energy density (*U*_d_) and efficiency (*η*) are drawing increasing research interest [2,3,4]. High *U*_d_ helps reduce the size and weight of electronics. High *η* benefits mitigate thermal dissipation in electronics, thus promoting their stability and lifespan. Generally, the storage energy density (*U*_e_), *U*_d_, and *η* of dielectric materials can be calculated from polarization–electric field (*P–E*) curves (Figure S1) by the following equations, respectively:

(1) $$U_{e}=\int_{0}^{P_{max}}EdP$$

(2) $$U_{d}=\int_{P_{max}}^{P_{r}}EdP$$

(3) $$\eta =U_{d}/U_{e}\times 100\%$$

where *E* is the applied electric field and *P* is the induced polarization, and *P*_max_ and *P*_r_ represent the maximum and remnant polarizations, respectively. For linear dielectrics, the maximum *U*_d_ (*U*_max_) is determined by the following Equation:

(4) $$U_{max}=0.5\varepsilon_{0}\varepsilon_{r}{E_{b}}^{2}$$

where *ε*_0_ is the vacuum permittivity, and *ε*_r_ and *E*_b_ are the dielectric constant and breakdown strength of dielectrics, respectively.

Polymer dielectrics play a critical role in the modern electrical industry, because of their high *E*_b_, light weight, and easy processing [5]. However, their low *U*_d_ limits their application prospects. Take the commercially available biaxially oriented polypropylene as an example, which exhibits a *U*_max_ of ~3 J/cm^3^ because of its low *ε*_r_ of ~2 [6,7]. High-*ε*_r_ inorganic nanofillers are usually introduced to polymer matrices to enhance their *ε*_r_, and hence their *U*_max_ [8,9,10]. By comparison, enhancing *E*_b_ is a more effective strategy to enhance *U*_max_ according to Equation (4). Many factors affect the *E*_b_ of polymer nanocomposites, such as the morphology and orientation state of fillers and the structure of nanocomposite films, among which the components or intrinsic properties of fillers are the most fundamental factor [11,12].

BaTiO_3_ (BT), as the best-known ferroelectric, is most frequently utilized as a filler because of its large *P*_max_ or *ε*_r_ [13,14,15]. However, its large *P*_max_ is usually accompanied by a large *P*_r_, resulting in a low *η*. Its large *ε*_r_ mismatch with polymer matrices and its relatively low *E*_b_ restrict the *E*_b_, and consequently the *U*_d_ enhancement, of polymer nanocomposites [16]. Compared with ferroelectric BT, forms of relaxor ferroelectric BT-Bi(M)O_3_ usually possess much slimmer *P*–*E* loops and higher *U*_max_ and *η* [17,18], and are attracting increasing attention in the field of energy storage ceramic dielectrics. Moreover, most of them display decreased *ε*_r_ and dielectric loss [19], which may be conducive to enhancing the *E*_b_ of polymer-based dielectric nanocomposites. However, very few of them have been reported as fillers.

On the basis of the aforementioned considerations, 0.6BaTiO_3_-0.4Bi(Mg_0.5_Ti_0.5_)O_3_ and 0.85BaTiO_3_-0.15Bi(Mg_0.5_Zr_0.5_)O_3_ nanofibers coated with highly insulating SiO_2_ (denoted as BT-BMT@SO_nfs and BT-BMZ@SO_nfs, respectively) were prepared as fillers in PVDF-based nanocomposites. Besides the components of fillers, the structures of nanocomposite films were also considered. Three kinds of nanocomposite films, single-layer BT-BMT@SO_nf/PVDF, single-layer BT-BMZ@SO_nf/PVDF, and sandwich-structured BT-BMZ@SO_nf/PVDF, were fabricated, and their dielectric and energy storage properties were investigated. Results show that BT-BMZ@SO_nfs and the sandwich structure favor the *E*_b_ enhancement of polymer nanocomposites. The sandwich-structured PVDF/6 wt% BT-BMZ@SO_nf within PVDF/PVDF nanocomposite films achieved a *U*_max_ of 11.1 J/cm^3^_,_ which is around 2.15 times that of pristine PVDF.

## 2. Materials and Methods

### 2.1. Materials

Barium hydroxide octahydrate (Ba(OH)_2_·8H_2_O), magnesium ethoxide (Mg(OC_2_H_5_)_2_), zirconium n-butoxide solution (80 wt% in n-butanol), PVP (M_w_ = 1,300,000), acetic acid, and ammonia solution (25–28 wt%) were purchased from the Aladdin Industrial Corporation (Shanghai, China). Bismuth acetate (Bi(CH_3_COO)_3_) was kindly provided by Hubei Xinkang Pharmaceutical & Chemical Co., Ltd. (Tianmen, China). Tetrabutyl titanate, acetyl acetone, and *N*,*N*-Dimethylformamide (DMF) were supplied by the Tianjin Kemiou Chemical Reagent Co., Ltd. (Tianjin, China). Ethanol absolute was supplied by the Sinopharm Chemical Reagent Co., Ltd. (Shanghai, China). Tetraethyl orthosilicate (TEOS) was bought from the Xilong Chemical Co., Ltd. (Shantou, China). PVDF powders were bought from Sigma-Aldrich (Shanghai, China).

### 2.2. Preparation of BT-Bi(M)O_3__nfs via Electrospinning

To prepare the BT-BMZ precursor, 8.5 mmol of Ba(OH)_2_·8H_2_O, 1.5 mmol of Bi(CH_3_COO)_3_, and 0.75 mmol of Mg(OC_2_H_5_)_2_ powders were dissolved in 9 mL of acetic acid and magnetically stirred for 30 min to obtain a transparent solution (A). Next, 8.5 mmol of tetrabutyl titanate and 0.75 mmol of zirconium n-butoxide were added into a solution containing 3 mL of ethanol absolute and 1 mL of acetyl acetone and stirred to form a yellowish transparent solution (B). Solution A was added to solution B drop by drop under continuous stirring, and then 6 g of 20 wt% PVP acetic solution was added to adjust the viscosity of the solution. The obtained transparent solution was further stirred for 12 h before use. The procedure for preparing BT-BMT precursor was almost the same as above procedure. The electrospinning process was performed under an applied electric field of 1.2 kV/cm. The distance between the needle and collector was fixed at 15 cm, and an injection rate of 1.0 mL/h was adopted. The collected nonwoven fabric was vacuum-dried at 80 °C for 12 h and subsequently calcined at 750 °C for 3 h to obtain crystalline BT-Bi(M)O_3__nfs.

### 2.3. Surface Coating of BT-Bi(M)O_3__nfs with SiO_2_

BT-Bi(M)O_3__nfs were coated with SiO_2_ (BT-Bi(M)O_3_@SO_nfs) via a modified Stöber method [20]. Typically, 0.75 g of BT-Bi(M)O_3__nfs were dispersed in a mixture of 600 mL of 2-propanol, 100 mL of deionized water, and 15 mL of 25–28 wt% ammonia solution. Next, 1.4 mL of TEOS was added into the suspension under vigorous stirring. The suspension was further mechanically stirred for 3 h to ensure the homogeneous coating of SiO_2_. The obtained BT-Bi(M)O_3_@SO_nfs were washed with deionized water 5 times and collected via suction filtration. Before use, they were vacuum-dried at 80 °C for 12 h.

### 2.4. Fabrication of BT-Bi(M)O_3_@SO_nf/PVDF Nanocomposite Films

BT-Bi(M)O_3_@SO_nfs were ultrasonically dispersed in DMF. PVDF was then added in proportion. The obtained mixtures were stirred for 24 h and degassed in vacuum before use. Single-layer BT-Bi(M)O_3_@SO_nfs/PVDF nanocomposite films were fabricated via a solution casting method. Sandwich-structured PVDF/x wt% BT-Bi(M)O_3_@SO_nfs/PVDF/PVDF nanocomposite films (denoted as 0-x-0) were prepared layer by layer through solution casting. PVDF/6 wt% 0.85BaTiO_3_-0.15Bi(Mg_0.5_Zr_0.5_)O_3_@SiO_2_ nanofiber/PVDF/PVDF nanocomposite films, the as-cast nanocomposite films, were then vacuum-dried at 80 °C for 12 h to remove DMF. Finally, the films were heated at 200 °C for 10 min, immediately quenched in an ice-water bath, and then peeled off of the quartz substrates and vacuum-dried at 60 °C for 12 h to remove residual moisture.

### 2.5. Finite Element Analysis of Electric Field Distribution

The local electric field distribution in the nanocomposite films was numerically simulated using COMSOL Multiphysics software (Version 5.3a). For convenience, the inner and outer diameters of BT-BMT@SO_nfs were set as 100 nm and 150 nm, respectively; and for BT-BMZ@SO_nfs they were set as 125 nm and 150 nm, respectively, so that their total diameters were equal. The length of the nanofibers was set as ~2.6 μm. The dielectric constants of BT-BMT, BT-BMZ, SiO_2_, and PVDF were set as 1300 [17,21], 900 [22,23], 4, and 9, respectively. An electric field of 300 MV/m was applied on the models.

### 2.6. Characterization

A scanning electron microscope (Nova Nano SEM 450, Hillsboro, OR, USA) and a transmission electron microscope (HT7700 EXALENS, Chiyoda City, Japan) were used to observe morphologies. An X-ray powder diffractometer with Cu Kα radiation (XRD, SmartLab 9KW, Rigaku, Tokyo, Japan) was used to identify phase structures. Raman spectroscopy was recorded on an inVia Qontor spectrometer (Gloucestershire, UK). Fourier-transform infrared (FTIR) spectroscopy was recorded using a FT/IR-6700 spectrophotometer (Tokyo, Japan). For the measurement of room-temperature dielectric and electrical properties, gold electrodes with a diameter of 3 mm were sputtered on both sides of nanocomposite films. Dielectric properties were tested using a precision impedance analyzer (Agilent Technologies 4294A, Santa Clara, CA, USA) at 1 Vrms. Breakdown strength was tested in silicone using a Changsheng 2674BX dielectric withstand voltage test system (Nanjing, China). The *P*–*E* loop was measured at 10 Hz using a Radiant Premier II ferroelectric test system (Los Angeles, CA, USA).

## 3. Results

**Figure 1:**
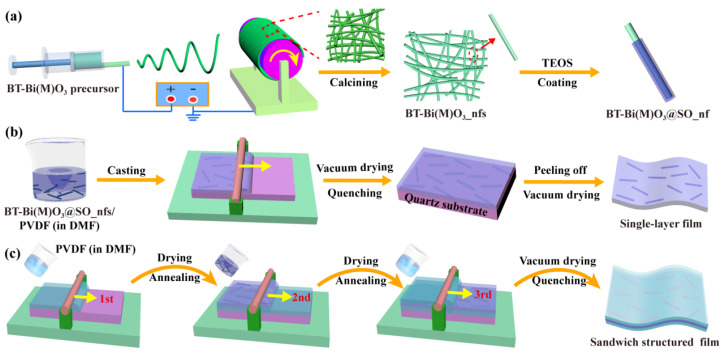
Schematic illustrations of preparing (**a**) BT-Bi(M)O_3_@SO_nfs, (**b**) single-layer, and (**c**) sandwich-structured BT-Bi(M)O_3_@SO_nf/PVDF nanocomposite films.

**Figure 2:**
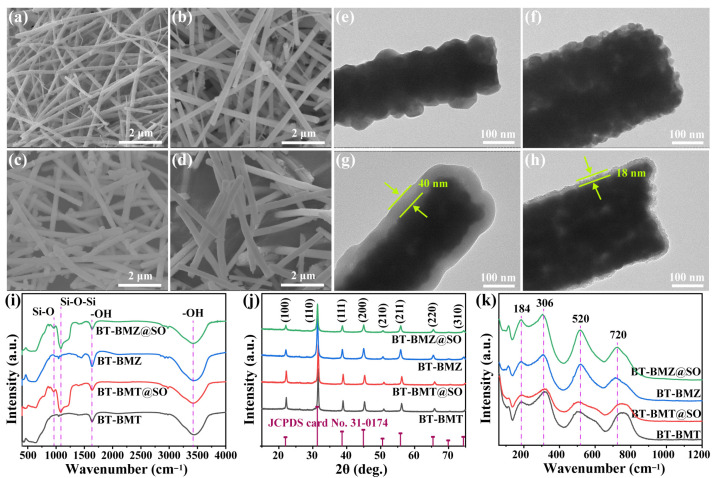
SEM images of (**a**) BT-BMT_nfs, (**b**) BT-BMZ_nfs, (**c**) BT-BMT@SO_nfs, and (**d**) BT-BMZ@SO_nfs; TEM images of (**e**) BT-BMT_nfs, (**f**) BT-BMZ_nfs, (**g**) BT-BMT@SO_nfs, and (**h**) BT-BMZ@SO_nfs; (**i**) FT-IR spectra, (**j**) XRD patterns, and (**k**) Raman spectra of these nanofibers.

BT-Bi(M)O_3__nfs were prepared via electrospinning, followed by calcining at 750 °C for crystallization (Figure 1a). Crystalline BT-Bi(M)O_3__nfs showed large aspect ratios (Figure 2a,b). BT-BMT_nfs showed a lower average diameter than BT-BMZ_nfs did; these were 184 nm and 267 nm, respectively (Figure S2a,b). To improve electric insulation properties, SiO_2_ was coated onto BT-Bi(M)O_3__nfs via a modified Stöber method [20]. After coating, the average diameters of BT-BMT@SO_nfs and BT-BMZ@SO_nfs increased to 277 nm and 318 nm, respectively (Figure 2c,d and Figure S2c,d). Thus, the calculated SiO_2_ shell thicknesses of BT-BMT@SO_nfs and BT-BMZ@SO_nfs were around 46 nm and 25 nm, respectively. TEM images clearly display the SiO_2_ coating layers on BT-BMT@SO_nfs and BT-BMZ@SO_nfs and verify their thickness differences (Figure 2e–h). FTIR spectra further identify the successful coating of SiO_2_ (Figure 2i). Compared with BT-Bi(M)O_3__nfs, two new bands at 960 cm^−1^ and 1087 cm^−1^ appeared in BT-Bi(M)O_3_@SO_nfs, which were assigned to the bending vibration of Si–OH and the tensile vibration of Si–O–Si, respectively. The highly insulating SiO_2_ layer enhanced the *E*_b_ of ceramic/polymer nanocomposites [24,25].

XRD patterns of the nanofibers are shown in Figure 2j, illustrating these materials as solid solutions with a perovskite structure. The diffraction peaks of BT-BMT_nfs are at higher angle positions than those of BT-BMZ_nfs, indicating their smaller unit cells. This result is consistent with previous studies, which found the expansion and shrinkage of unit cells in BT-BMZ and BT-BMT compared with BT (JCPDS card No. 31-0174), respectively [17,23]. The XRD peaks of BT-Bi(M)O_3_@SO_nfs were the same as those of corresponding BT-Bi(M)O_3__nfs, indicating the SiO_2_ layers were amorphous. Splitting of the (002) peak could not be observed in all samples, indicating the absence of non-cubic distortions. Raman measurement was performed to further study their local structures (Figure 2k). The absence of a 270 cm^−1^ peak and the existence of a 720 cm^−1^ peak verified their local short-range order structures rather than long-range order structures. Compared with BT-BMZ_nfs, Raman peaks located at 306 cm^−1^, 520 cm^−1^, and 720 cm^−1^ broadened in BT-BMT_nfs. This was because many more ions like Bi^3+^ and Mg^2+^ entered the BT crystal lattices, resulting in the short-range polarization mismatch, and consequently leading to the broadening of Raman peaks. BT-BMT and BT-BMZ ceramics without long-range ferroelectricity ordering usually show relaxor ferroelectricity [17,23].

**Figure 3:**
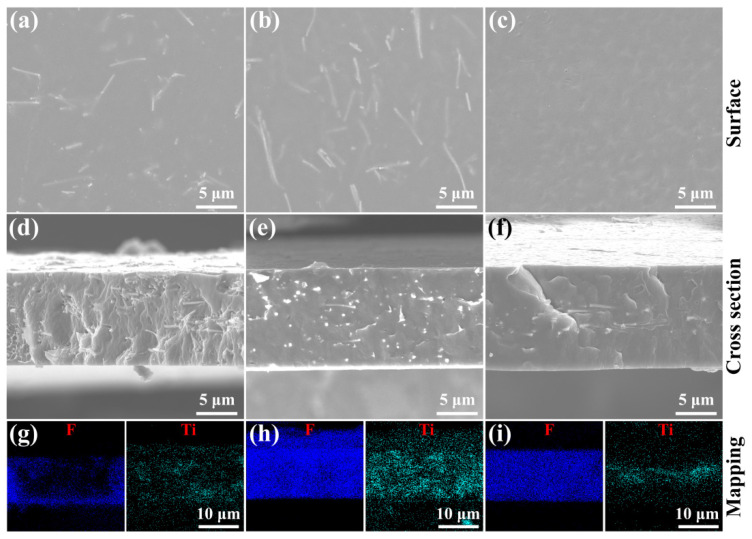
Surface SEM images, cross-sectional SEM images, and element mappings of (**a**,**d**,**g**) 8 wt% BT-BMT@SO_nf/PVDF, (**b**,**e**,**h**) 8 wt% BT-BMZ@SO_nf/PVDF, and (**c**,**f**,**i**) 0-8-0 wt% BT-BMZ@SO_nf/PVDF nanocomposite films.

Single-layer BT-Bi(M)O_3_@SO_nf/PVDF nanocomposite films were prepared via a facile solution casting method (Figure 1b). Sandwich-structured BT-BMZ@SO_nf/PVDF nanocomposite films were prepared layer by layer through solution casting (Figure 1c). Their thicknesses were well controlled by the scraper, ranging from 11–16 μm (Figure 3d–f). For single-layer films, BT-Bi(M)O_3_@SO_nfs could be observed in both surface and cross-sectional SEM images (Figure 3a,b,d,e). Moreover, their element mappings show that Ti and F elements shared similar distribution states (Figure 3g,h). These results reveal that BT-Bi(M)O_3_@SO_nfs homogeneously disperse in the whole single-layer films. For the sandwich-structured film, BT-BMZ@SO_nfs could only be observed in cross-sectional SEM images (Figure 3c,f and Figure S3). Moreover, the Ti element was predominantly distributed in the middle layer of these films, contrasting with the distribution state of the F element (Figure 3i). These results indicate that BT-BMZ@SO_nfs dispersed only in the middle layer of sandwich-structured films. In all these films, BT-Bi(M)O_3_@SO_nfs mainly oriented in the in-plane directions. No obvious defects, such as cracks and voids, could be observed; these features are beneficial breakdown properties.

**Figure 4:**
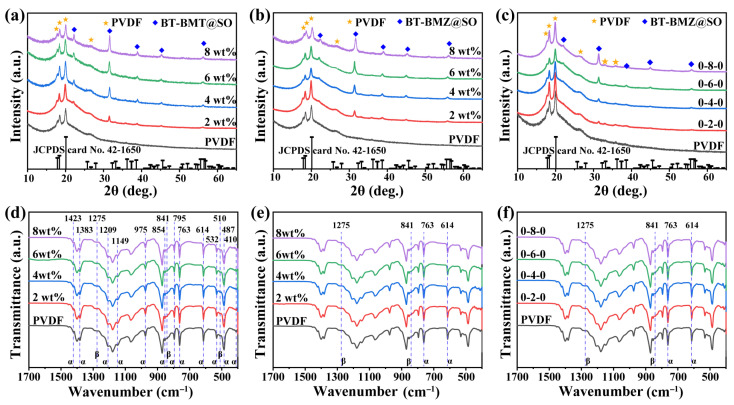
XRD patterns of (**a**) single-layer BT-BMT@SO/PVDF, (**b**) single-layer BT-BMZ@SO/PVDF, and (**c**) sandwich-structured BT-BMZ@SO/PVDF nanocomposite films; and FTIR spectra of (**d**) single-layer BT-BMT@SO/PVDF, (**e**) single-layer BT-BMZ@SO/PVDF, and (**f**) sandwich-structured BT-BMZ@SO/PVDF nanocomposite films.

XRD patterns (Figure 4a–c) and FTIR spectra (Figure 4d–f) of these nanocomposite films were also characterized to study their crystalline properties. For pristine PVDF, XRD peaks appeared at 2θ = 17.8°, 18.5°, 20.0°, and 26.6°, and were assigned to the (100), (020), (110), and (021) lattice planes of the α phase (JCPDS card No. 42-1650), respectively [26]. XRD peaks for all these nanocomposite films can be classified into two parts; i.e., those from α-phase PVDF and those from BT-Bi(M)O_3_@SO_nfs. Apart from the α phase, FTIR spectra clarified the existence of the β phase of PVDF in all samples. Absorption bands at 1423 cm^−1^, 1383 cm^−1^, 1209 cm^−1^, 1149 cm^−1^, 975 cm^−1^, 854 cm^−1^, 795 cm^−1^, 763 cm^−1^, 614 cm^−1^, 487 cm^−1^, and 410 cm^−1^ were assigned to the nonpolar α-phase PVDF [26]. And the absorption bands at 1275 cm^−1^, 841 cm^−1^, and 510 cm^−1^ were assigned to the polar β-phase PVDF. Absorbencies at 763 cm^−1^ and 841 cm^−1^ are usually utilized to calculate the relative amounts of these two phases according to the Lambert–Beer law [27,28]. The intensity of the band at 763 cm^−1^ was much stronger than that at 841 cm^−1^, indicating that the crystalline PVDF was dominated by the α phase. This result may account for the absence of XRD peaks of β-phase PVDF. Meanwhile, the intensities of these two bands remained almost unchanged as the filler content changed, suggesting BT-Bi(M)O_3_@SO_nfs had little effect on the relative contents of the α and β phases of crystalline PVDF.

Figure S4 shows the frequency-dependent dielectric properties of the nanocomposite films. Both the *ε*_r_ and dielectric loss (tan *δ*) of all films slightly decreased as the frequency increased from 10^2^ Hz to 10^4^ Hz, which was attributed to the suppressed interfacial polarization that occurred at elevated frequencies [29,30]. As the frequency further increased from 10^4^ Hz to 10^6^ Hz, the *ε*_r_ sharply decreased and the dielectric loss sharply increased, which was ascribed to the dipolar relaxation of PVDF at high frequencies [31].

**Figure 5:**
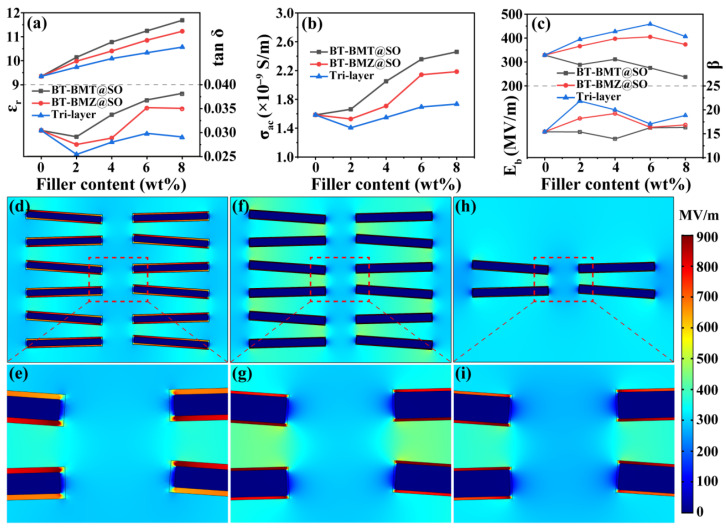
(**a**) Dielectric constants and dielectric losses of the nanocomposite films at 1 kHz; (**b**) AC conductivities of the nanocomposite films at 1 kHz; (**c**) breakdown strengths and Weibull moduli of the nanocomposite films; finite element simulation of local electric field distribution for (**d**,**e**) single-layer BT-BMT@SO_nf/PVDF, (**f**,**g**) single-layer BT-BMZ@SO_nf/PVDF, and (**h**,**i**) sandwich-structured BT-BMZ@SO_nf/PVDF nanocomposite films.

Dielectric properties of the nanocomposite films at 1 kHz are summarized in Figure 5a. The *ε*_r_ of the three kinds of samples increased monotonically with increasing filler content. This result is in line with the effective medium theory that proposed that the introduction of high-*ε*_r_ ceramic nanofillers into polymer matrices could enhance their *ε*_r_ [8]. With the same filler content, the *ε*_r_ and dielectric loss of single-layer BT-BMZ@SO_nf/PVDF nanocomposite films were lower than those of single-layer BT-BMT@SO_nf/PVDF nanocomposite films. This result is mainly ascribed to the fact that BT-BMZ ceramic has lower *ε*_r_ and dielectric loss than BT-BMT ceramic does [17,21,22,23]. The sandwich-structured BT-BMZ@SO_nf/PVDF nanocomposite films possessed the lowest *ε*_r_ because of their relatively low total filler content, which was around one third of that of corresponding single-layer nanocomposite films. Meanwhile, they possessed the lowest dielectric loss, which can mainly be attributed to the barrier effect of the sandwich structure [32,33]. Alternating current conductivities (*σ*_ac_) of the nanocomposite films were also tested (Figure S5). The sandwich-structured BT-BMZ@SO_nf/PVDF nanocomposite films showed the lowest values, at 1 kHz, among the three kinds of samples (Figure 5b).

Breakdown strength is a crucial parameter in determining the energy storage capability and operative electric field of dielectric materials. Breakdown properties of the nanocomposite films were evaluated by a two-parameter Weibull distribution function described as follows:

(5) $$P\left(E\right)=1-exp(-{\left(E/E_{b}\right)}^{\beta })$$

where *P*(*E*) refers to the cumulative probability of electric failure, *E* is the measured breakdown strength, *E*_b_ is the characteristic breakdown strength, and the Weibull modulus *β* evaluates the breakdown reliability. The Weibull plots and fitted lines of the measured breakdown strength of the nanocomposite films are shown in Figure S6. Their *E*_b_ and *β* values are summarized in Figure 5c. With the same filler content, the single-layer BT-BMT@SO_nf/PVDF and sandwich-structured BT-BMZ@SO_nf/PVDF nanocomposites possessed the lowest and highest *E*_b_, respectively. Meanwhile, they also showed the lowest and highest *β* values, respectively, indicating the worst and the best reliability. That the sandwich-structured BT-BMZ@SO_nf/PVDF nanocomposites achieved the best overall breakdown properties might be ascribed to their lowest dielectric loss and *σ*_ac_.

The electric field distribution in three kinds of nanocomposite films was simulated using the finite element method (Figure 5d–i). The electric field in the middle layer of sandwich-structured BT-BMZ@SO_nf/PVDF films was less concentrated than that in the corresponding area of single-layer BT-BMZ@SO_nf/PVDF films (Figure 5f–i). This was because the middle nanocomposite layer had a higher *ε*_r_ than the outer pristine PVDF layers did, and thus bore a lower average electric field. The middle layer with alleviated electric field concentration could act as barrier for electrical treeing at the layer interfaces [31], resulting in enhanced *E*_b_ of sandwich-structured BT-BMZ@SO_nf/PVDF films.

**Figure 6:**
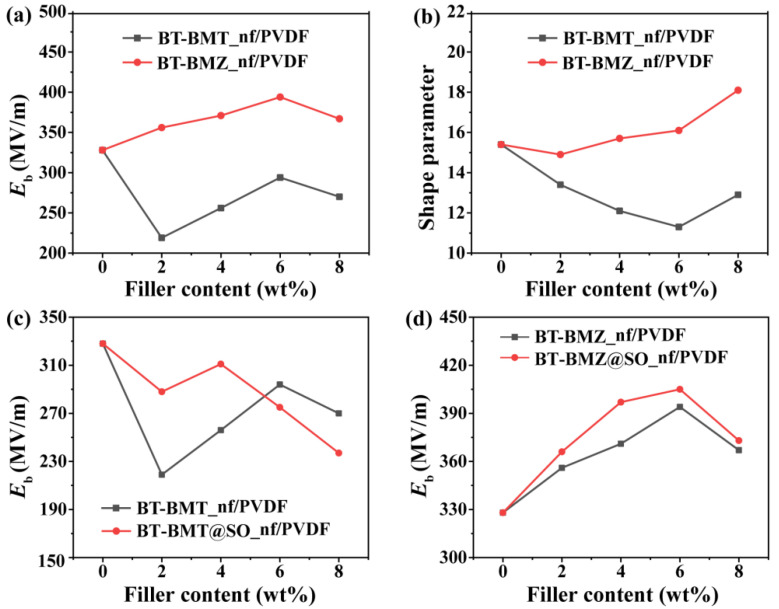
Comparisons of (**a**) characteristic breakdown strengths and (**b**) shape parameters between BT-BMT_nf/PVDF and BT-BMZ_nf/PVDF nanocomposite films; and comparisons of characteristic breakdown strengths between (**c**) BT-BMT_nf/PVDF and BT-BMT@SO_nf/PVDF and (**d**) BT-BMZ_nf/PVDF and BT-BMZ@SO_nf/PVDF nanocomposite films.

Among the three kinds of samples, the local electric field concentration phenomenon in the single-layer BT-BMT@SO_nf/PVDF nanocomposite film was the weakest (Figure 5d,e), which was ascribed to the thickest SiO_2_ shell. However, the BT-BMT@SO_nf/PVDF nanocomposite films had the lowest *E*_b_. To evaluate the effect of the BT-Bi(M)O_3_ core and SiO_2_ shell on breakdown properties, the breakdown strengths of BT-BMT_nf/PVDF and BT-BMZ_nfs/PVDF nanocomposite films were measured (Figure S7). Results show that the BT-BMT_nf/PVDF nanocomposite films had a much lower *E*_b_ and Weibull moduli than those of corresponding BT-BMZ_nf/PVDF nanocomposite films (Figure 6a,b). Results also show that the SiO_2_ shell could indeed enhance the *E*_b_ of BT-Bi(M)O_3_@SO_nf/PVDF nanocomposite films (Figure 6c,d). However, the breakdown properties were primarily determined by the BT-Bi(M)O_3_ core rather than the SiO_2_ shell.

According to the above analysis, it can be concluded that the intrinsic properties of nanofillers are the determining factors of the *E*_b_ of polymer-based nanocomposites, and that the SiO_2_ coating and film structure design are effective strategies to enhance their *E*_b_.

**Figure 7:**
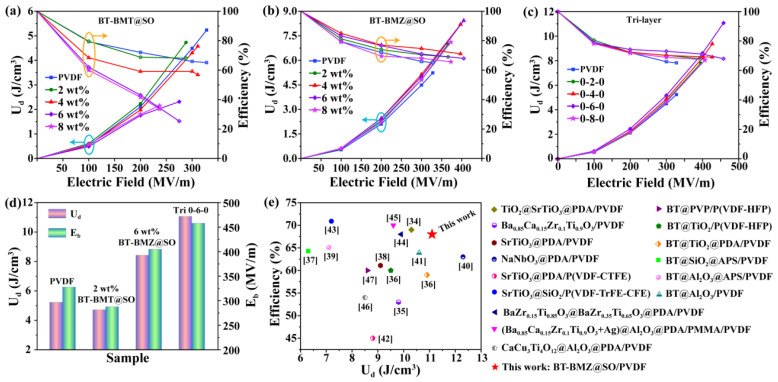
Discharge energy densities and efficiencies of three kinds of nanocomposite films: (**a**) single-layer BT-BMT@SO_nf/PVDF, (**b**) single-layer BT-BMZ@SO_nf/PVDF, and (**c**) sandwich-structured BT-BMZ@SO_nf/PVDF. (**d**) Comparison of maximum discharge energy densities and corresponding efficiencies of three kinds of nanocomposite films. (**e**) Comparison of energy storage properties between 0-6-0 BT-BMZ@SO_nf/PVDF nanocomposite films and recently reported PVDF-based nanocomposites with 1D fillers [34,35,36,37,38,39,40,41,42,43,44,45,46,47].

*P–E* curves of the nanocomposite films were measured to evaluate their energy storage properties (Figure S8). For all samples, the increasing electric field induced increased *P*_max_ and *P*_r_, resulting in increased *U*_e_, but decreased *η* (Figure 7a–c). Meanwhile, *U*_d_ also increased with the increasing electric field. At the same electric field, the *P*_r_ of the single-layer BT-BMT@SO_nf/PVDF nanocomposite films obviously increased when the filler content exceeded 2 wt%. As a result, their *η* sharply decreased, resulting in decreased *U*_d_ (Figure 7a). By comparison, the single-layer and sandwich-structured BT-BMZ@SO_nf/PVDF nanocomposite films showed much slimmer *P–E* curves, and hence higher *η* (Figure 7b,c). Although those films with different filler contents showed small differences in *U*_d_ under the same electric fields, they showed large differences in *U*_max_ because of *E*_b_ differences and the induced *P*_max_ differences (Figure S8).

The 2 wt% BT-BMT@SO_nf/PVDF, 6 wt% BT-BMZ@SO_nf/PVDF, and 0-6-0 BT-BMZ@SO_nf/PVDF nanocomposite films possessed the largest *U*_max_ among the three kinds of nanocomposites, respectively. Their *U*_max_ and *E*_b_ are summarized in Figure 7d. The *U*_max_ of pristine PVDF and these three nanocomposite films were 5.2, 4.7, 8.4, and 11.1 J/cm^3^, with ηs of 65.1%, 68.1%, 68.0%, and 68.0%, respectively. Their differences in η were subtle, thus their large *U*_max_ differences can mainly be attributed to *E*_b_ differences and induced *P*_max_ differences. Their *E*_b_ were 328, 288, 405, and 458 MV/m, with induced *P*_max_ of 4.7, 4.6, 6.1, and 7.0 μC/cm^2^, respectively. A comparison of energy storage properties between the 0-6-0 BT-BMZ@SO_nf/PVDF nanocomposite films and recently reported PVDF-based nanocomposites with 1D fillers is depicted in Figure 7e [16,34,35,36,37,38,39,40,41,42,43,44,45,46,47]. It can be seen that the 0-6-0 BT-BMZ@SO_nf/PVDF nanocomposite films show better comprehensive energy storage properties than most representative nanocomposites.

## 4. Conclusions

Three kinds of polymer nanocomposites, single-layer BT-BMT@SO_nf/PVDF, single-layer BT-BMZ@SO_nf/PVDF, and sandwich-structured BT-BMZ@SO_nf/PVDF, were fabricated via a solution casting method. The comparison of their energy storage properties proves that *E*_b_ is a critical parameter for enhancing the *U*_max_ of polymer nanocomposites. The combination of simulated results and experimental results shows that a SiO_2_ coating can improve the breakdown properties of single-layer BT-Bi(M)O_3__nf/PVDF nanocomposites, and that the components or the intrinsic properties of BT-Bi(M)O_3_ are the determining factors for their *E*_b_. Although constructing sandwich-structured films sacrificed their *ε*_r_ to some extent, it was effective in enhancing their *E*_b_, and consequently their *U*_d_. As a result, the sandwich-structured 0-6-0 BT-BMZ@SO_nf/PVDF nanocomposite films achieved the best overall energy storage performance. Their *U*_d_ reached 11.1 J/cm^3^ at an *E*_b_ of 458 MV/m, which are ~2.15 and ~1.40 times those of pristine PVDF. Based on these results, two points should be emphasized to enhance the *U*_d_ of BaTiO_3_-Bi(M)O_3_@SiO_2_/PVDF nanocomposites: one is that the component of the nanofillers is a determining factor; the other is that the structural design of nanocomposite films is an effective strategy.

## Data Availability

Data are contained within the article and Supplementary Materials.

## Supplementary Materials

The following supporting information can be downloaded at: https://www.mdpi.com/article/10.3390/nano15080569/s1, Figure S1: Schematic illustration of calculating storage energy density (*U*_e_), discharge energy density (*U*_d_), and efficiency (*η*) of non-linear dielectrics from a *P*–*E* loop; Figure S2: Diameter distributions of (a) BT-BMT_nfs, (b) BT-BMZ_nfs, (c) BT-BMT@SO_nfs, and (d) BT-BMZ@SO_nfs; Figure S3: Cross-sectional SEM images of (a) 0-2-0, (b) 0-4-0, and (c) 0-6-0 sandwich-structured BT-Bi(M)O_3_@SO_nf/PVDF nanocomposite films; Figure S4: Frequency-dependent dielectric constants and dielectric losses of (a) single-layer BT-BMT@SO_nf/PVDF, (b) single-layer BT-BMZ@SO_nf/PVDF, and (c) sandwich-structured BT-BMZ@SO_nf/PVDF nanocomposite films; Figure S5: Alternating current conductivities of (a) single-layer BT-BMT@SO_nf/PVDF, (b) single-layer BT-BMZ@SO_nf/PVDF, and (c) sandwich-structured BT-BMZ@SO_nf/PVDF nanocomposite films; Figure S6: Weibull plots of breakdown strength of (a) single-layer BT-BMT@SO_nf/PVDF, (b) single-layer BT-BMZ_nf@SO_nf/PVDF, and (c) sandwich-structured BT-BMZ@SO_nf/PVDF nanocomposite films; Figure S7: Weibull plots of breakdown strength of (a) BT-BMT_nf/PVDF and (b) BT-BMZ_nf/PVDF nanocomposite films; Figure S8: *P–E* loops of (a) PVDF, (b–e) single-layer BT-BMT@SO_nf/PVDF, (f–i) single-layer BT-BMZ@SO_nf/PVDF, and (j–m) sandwich-structured BT-BMZ@SO_nf/PVDF nanocomposite films under different electric fields.
