# Supplementary material for: Enhancing Energy Density of BaTiO3-Bi(M)O3@SiO2/PVDF Nanocomposites via Filler Component Modulation and Film Structure Design
Source: Nanomaterials (Basel). 2025 Apr 8;15(8):569. doi: 10.3390/nano15080569 (PMC12029639; doi:10.3390/nano15080569)
Supplement: Supplementary file 1 [file nanomaterials-15-00569-s001.zip › nanomaterials-3531663-supplementary.pdf]

Supplementary Material

Enhancing energy density of $BaTiO_3$-$Bi(M)O_3$@SiO_2/PVDF nanocomposites via filler component modulation and film structure design

Jin Hu[1] *and Fangfang Liu*[2]*

[1] State Key Laboratory of Fine Chemicals, Dalian University of Technology, Dalian 116024, China
[2] College of Science, National University of Defense Technology, Changsha 410073, China
* Correspondence: liufangfang@nudt.edu.cn

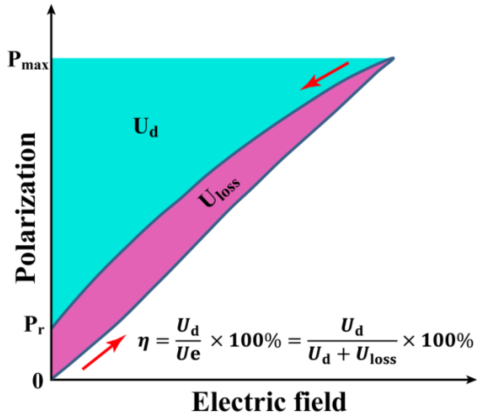

Figure S1. Schematic illustration of calculating storage energy density (U_e), discharge energy density (U_d) and efficiency (η) of non-linear dielectrics from a *P*-*E* loop.

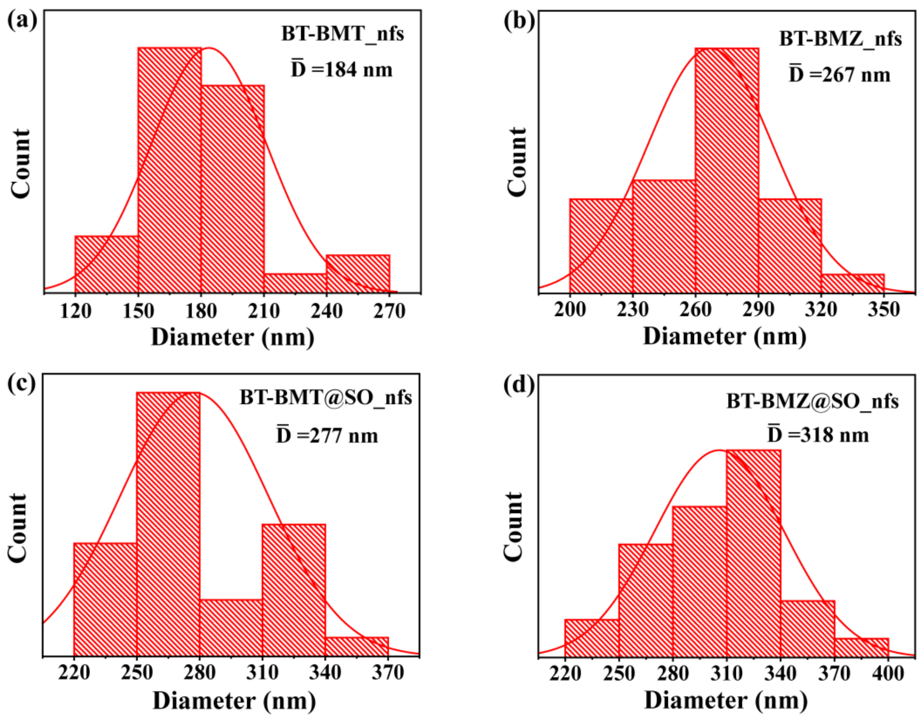

Figure S2. Diameter distributions of (a) BT-BMT_nfs, (b) BT-BMZ_nfs, (c) BT-BMT@SO_nfs, and (d) BT-BMZ@SO_nfs.

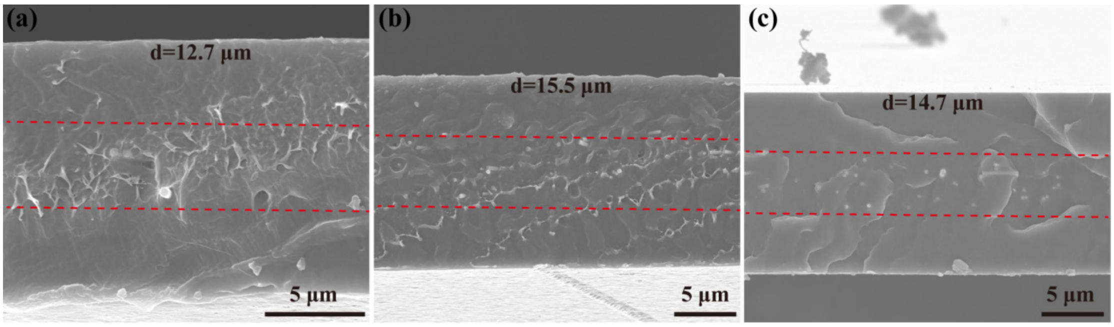

Figure S3. Cross-sectional SEM images of (a) 0-2-0, (b) 0-4-0 and (c) 0-6-0 sandwich-structured BT-Bi(M)O_3@SO_nf/PVDF nanocomposite films.

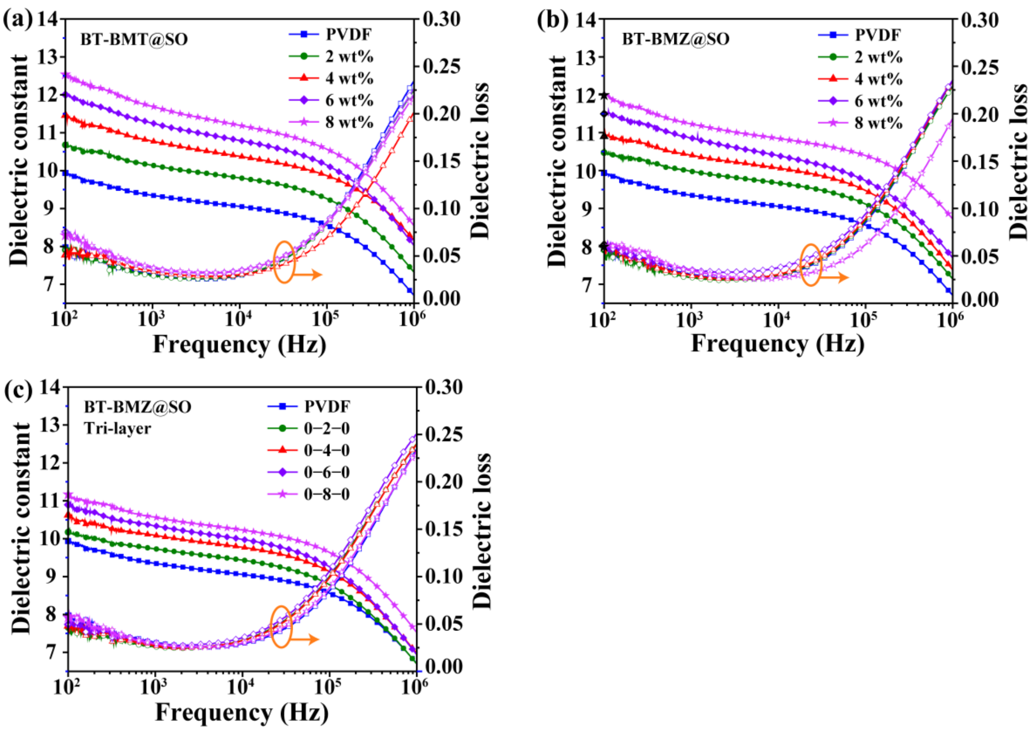

Figure S4. Frequency-dependent dielectric constants and dielectric losses of (a) single-layer BT-BMT@SO_nf/PVDF, (b) single-layer BT-BMZ@SO_nf/PVDF, and (c) sandwich-structured BT-BMZ@SO_nf/PVDF nanocomposite films.

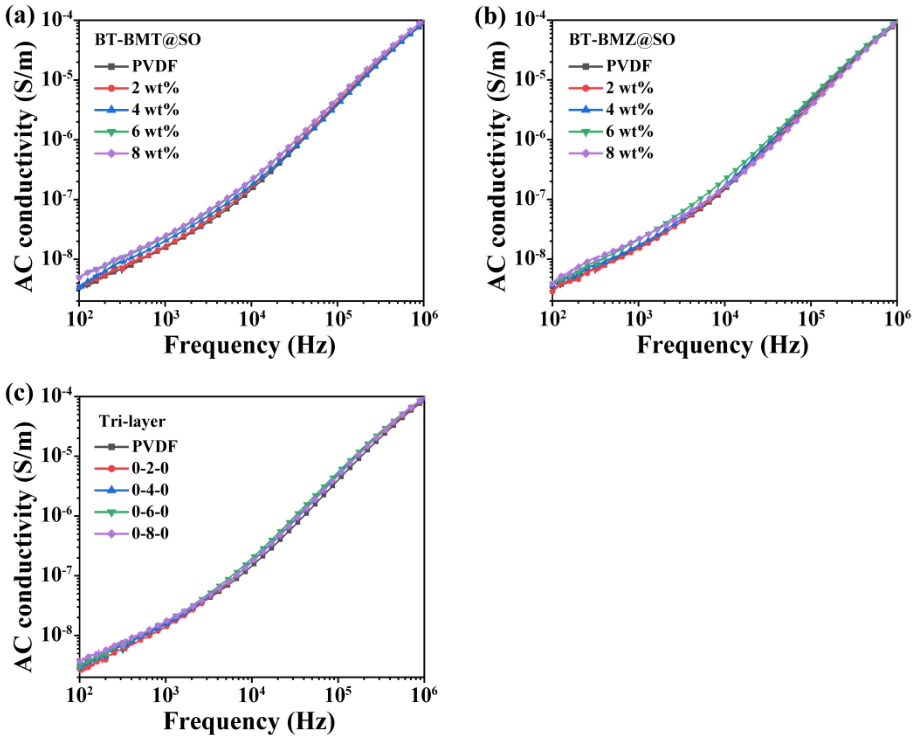

Figure S5. Alternating current conductivities of (a) single-layer BT-BMT@SO_nf/PVDF, (b) single-layer BT-BMZ@SO_nf/PVDF, and (c) sandwich-structured BT-BMZ@SO_nf/PVDF nanocomposite films.

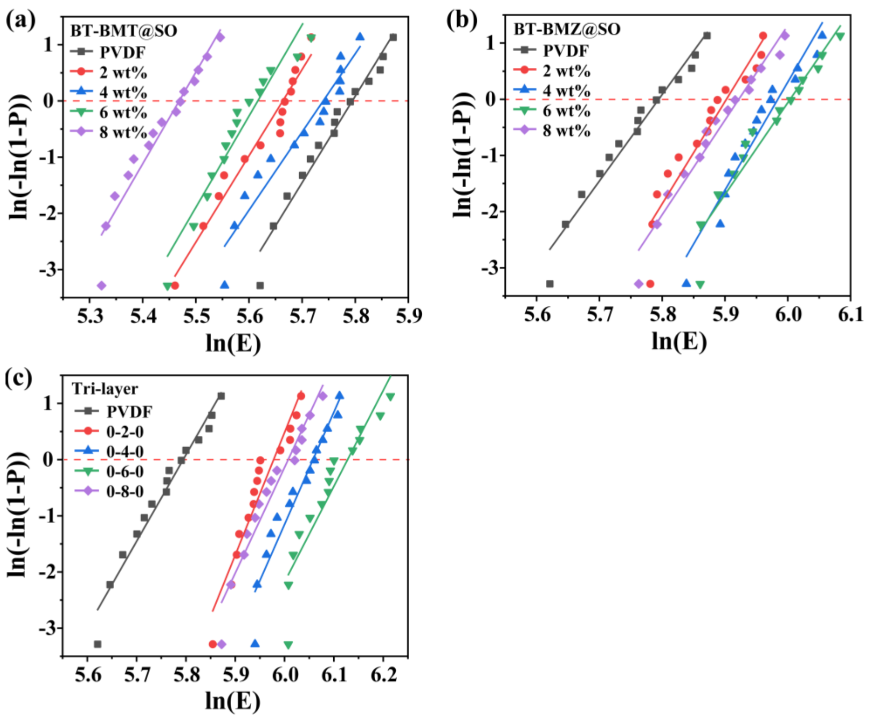

Figure S6. Weibull plots of breakdown strength of (a) single-layer BT-BMT@SO_nf/PVDF, (b) single-layer BT-BMZ_nf@SO_nf/PVDF, and (c) sandwich-structured BT-BMZ@SO_nf/PVDF nanocomposite films.

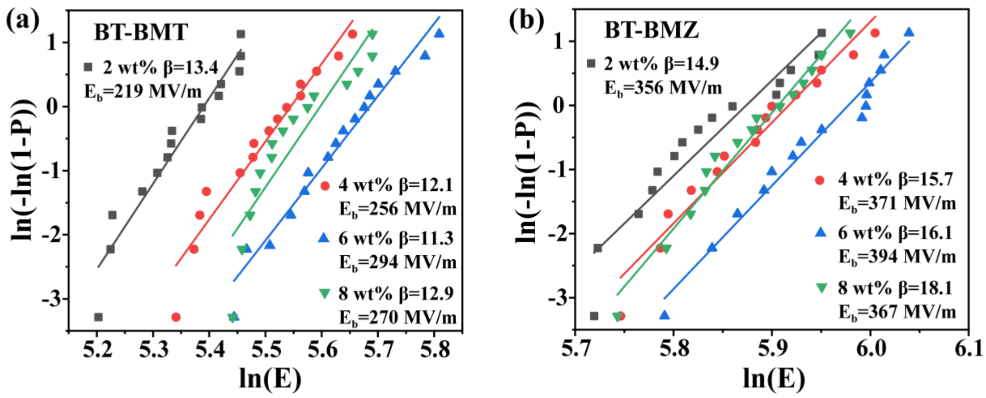

Figure S7. Weibull plots of breakdown strength of (a) BT-BMT_nf/PVDF and (b) BT-BMZ_nf/PVDF nanocomposite films.

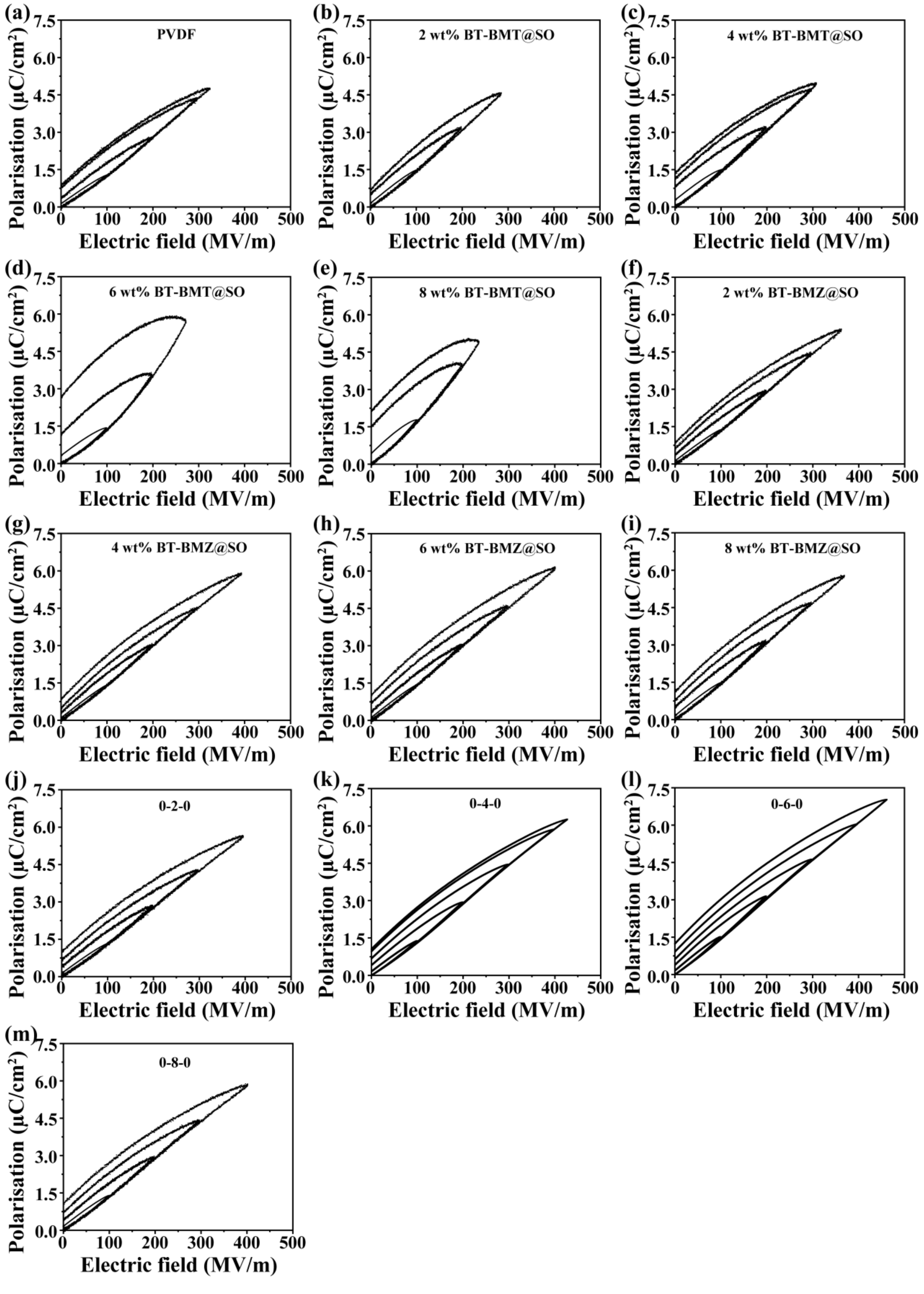

Figure S8. *P-E* loops of (a) PVDF, (b–e) single-layer BT-BMT@SO_nf/PVDF, (f–i) single-layer BT-BMZ@SO_nf/PVDF, and (j–m) sandwich-structured BT-BMZ@SO_nf/PVDF nanocomposite films under different electric fields.
